# Service and Self-Surrender

**Published:** 1921-01-29

**Authors:** Berkeley Moynihan

**Affiliations:** K.C.M.G.


### "THE MOST GENTLE PROFESSION."

### Service and Self-Surrender.

Address by SIR BERKELEY MOYNIHAN, K.C.M.G., C.B., F.R.C.S.*

The memory of unpleasing things is very tenacious. A century ago an operation was attended by a variety of circumstances calculated to arouse dismay and keen anxiety in every heart; and to-day the very word "operation" sends a shudder through many of those who learn that it is to be their early destiny. And this dread continues, though you and I know that an operation is an act of gentlest mercy, guided and determined by the utmost skill, inspired and controlled at every stage by compassion for the sufferer. To the word "nurse" a similar obloquy still attaches. Even now the word conjures up in some minds the picture of a bibulous and crapulous hag, unversed in the simplest rudiments of her art and indifferent and insensible to the needs of others. Her picture has been drawn by many hands; it was coarsely exaggerated, no doubt, even in Dickens' day, but the recollection of it is still fresh in many minds, though you and I know that the nurse of to-day is one of the most gracious and most competent of women, and that the profession of nursing now attracts the best type of womanhood that this country can produce.

### Difficulties Encountered in Private Nursing.

But those of you who leave this hospital to go out into the world to nurse must not be surprised if you find that you are not welcomed with that open, eager enthusiasm to which your training and your experience will fully entitle you. In your hospital life you are the despots, most merciful despots it is true, of all your patients, who conform with no word of denial or contumacy to all that is demanded of them or imposed upon them. They accept without question the discipline of the hospital and the beneficent rule of its officers. Of your work in private much will have to be done in the homes of your patients. Happily the day is almost past when an operation of any magnitude has to be performed in the unsuitable surroundings of a private house, with all the makeshifts and dangers inseparable from such work. But the convalescence of surgical cases and the whole course of a grave medical illness will be passed in a private house to whose rules and customs you will be expected to conform, in which, however great your competence and however congenial your society, you are an intruder. You will be compelled, looking only to your patient's welfare, to intervene between him and his friends, often running counter to their wishes and their normal practice as you shelter him from their well-meant but harmful attentions. It will be one of your many testing times, when you will require all the gifts, all the tact, and all the accomplishments that your natural aptitude, or your long training, have conferred upon you.

### Learn to Use Knowledge Justly.

How are you to fit yourself for such tasks, or to become competent to undertake with highest success all the manifold and arduous responsibilities that lie ahead of you? First, you will need knowledge. To gain it in an adequate degree you will require intellectual powers of no mean order and industry above the average. There is much to be learnt of anatomy, of physiology, of medicine, and of surgery; of the principles which underlie the technical work you will daily practise. You will have to avoid the little knowledge which is dangerous by delving as deeply as you can into those things which apply most nearly to your own tasks. It is better to learn intimately the relevant matters than to have a smattering of many things that it is not within your strict province to know. But knowledge which is within the reach of everyone who truly seeks it will avail you little unless it leads you along the way to wisdom. Wisdom implies the timely and rightful application of knowledge. Knowledge may even be a pitfall or an encumbrance unless you learn to use it justly. To gain wisdom is of all tasks in life the most difficult, and it is certainly no less arduous in nursing than in many other of life's activities. You will be foiled and rebuffed and disheartened, not once but many times, as you toil earnestly after it, for the application of the truths you have learnt may be so diverse, the reactions so unexpected and perplexing, and the personal aspects of them so capricious, that you may think of wisdom as Fracastorius did of the beating of the heart—that it "is so difficult as only to be comprehended by God."

You will have duties, fewer than they formerly were, which may appear menial or degrading, and they will sometimes need to be carried out upon those who are the mere wreckage of humanity. But drudgery may be a blessed thing, and you may derive consolation from the remembrance of One who thought it no ill task to wash the feet of the humblest of people. And you will perhaps day after day, especially in your early years, be almost dead with fatigue, embittered by the disappointments of a case that has gone wrong, or wounded by a rebuke that has escaped from the lips of someone as weary and disheartened as yourself. Yet all the time you must show your best side, for you cannot give real help to others if you seem careworn or dejected. You must learn to bring an air of pleasure to the pursuit of duty. And so by degrees you will learn that it is not only knowledge, or even wisdom, but also, and chiefly, character that counts. You will learn to deal faithfully, stubbornly, and with untiring zeal with all your difficulties, and the word "trouble" will vanish from your vocabulary. No patient can ever cause you "trouble" if you remember that what is a daily and perhaps monotonous event to you is the great event and perhaps the sternest trial of a lifetime to him. Your patient's needs are your opportunity.

### The Noblest Function of Man.

You will soon divine the great secret that in many patients who are seriously ill the restraints which adult life impose upon us all, fall away. The qualities of childhood again emerge; there is a trustful dependence upon others; there is great need of sympathy and understanding; there may be a little petulance, a little fretfulness, a querulous demand for many things unsuitable. You may need great patience, infinite gentleness, unfailing forbearance, if you are to read your patient aright, and to serve him to the utmost of your capacity. Service is the noblest function of man. And to render the highest service you must attune yourself spiritually with your patients, so that you may read their hearts, discover their motives, divine their impulses, and lead them at last to realise that you stand loyally behind

* Delivered at the Annual Prize Distribution to the Nursing Staff of the Leeds General Infirmary, January 21, 1921.

**"The Most Gentle Profession."**—*(continued)*.

them, or beside them, to help them, not over against them to thwart them. And at all times you must keep reticence. Many secrets that have perhaps been most jealously guarded will be disclosed to you, and many of the most sacred mysteries will be revealed. You will preserve an inviolable silence. For taciturnity is an ornament, and in silence there is security; if you repent once of your silence you will repent ten times of your speech. You will find help from the 39th Psalm, "I will take heed to my ways that I offend not in my tongue. I will keep my mouth as it were with a bridle." To chatter of those intimate things you learn under the seal of the confessional as you work is a degradation of your calling. Gossip tainted with slander is the last and meanest infirmity of empty minds.

THE NURSE'S OFFICE.

Such are some of the qualities required by a nurse, and this then is the nurse's office. To be ready in all emergency, quick and competent in action, courteous in speech, considerate in thought; a comfort in hours of sorrow, an inspiration and encouragement in times of gloom; to give ease to many a weary body and solace to many a troubled heart; to lift with strong and gentle hands a heavy load of anguish from those who falter and stumble in despair. It is to be a beacon of hope, a rock of refuge and a tower of strength.

If your attainments are these and your work of this high order, you are members of a profession than which none is gentler or nobler. Your watchwords become "service and self-surrender." You do not seek reward or selfish gain. Your work will be done in a professional spirit; it will be done, not in the most meagre way for the utmost gain, but with all the energy and truth that you can put into it. The true rewards of honest work are neither to be seen nor handled, they are not measured by a gold standard nor by any material result. They are not acclaimed by the applause of the crowd. They lie within you; in your own knowledge that you have done your best, that you have striven to reach your own standard of your highest powers. You will often, perhaps always, fail to reach your own ideal; but be comforted. Ideals are not for attainment, but for pursuit.

RECOGNITION OF EFFICIENCY.

If you enter a profession and become adept and worthy members of it you should receive the proper recognition to which your work entitles you. The time is now ripe, in my opinion, for your acceptance by some academic body which shall control the training and direct the teaching of the nursing profession, and in due time confer authority by licence, or diploma, or degree, upon those who have attained the standard of efficiency that is considered adequate. Your work, whether regarded as an intellectual task or as a technical accomplishment demanding the exercise of fine craftsmanship, fully entitles the nursing profession to make such a demand as this. If such a recognition comes, then will follow a result I have long desired to see. There will be a grading of nurses by qualification as there is a grading of medical men. It is, I think, just as necessary that a sister in charge of a ward, a theatre sister, or a matron in a teaching hospital should bear evidence in her qualification of longer study and more careful training as it is in the case of the medical staff of a teaching hospital. Until some system of supervision of the training of all who may call themselves nurses and of the registration and qualification by diploma or degree is introduced, the nursing profession will not be cleansed from those impurities which still unhappily attach to it.

THE HONOUR OF THE TRAINING SCHOOL.

In Leeds you are fortunate in your school. The training through which you pass here is as long, as arduous, as strict as it is anywhere in the world. And the honour of your school is of the highest; it has been created, maintained, and increased by the great multitude of your predecessors. Remember that you all carry with you, wherever you go, the honour and good repute of your school. Every one among you can add to that store of honour or detract from it. Leeds will be judged by your work and by your demeanour. And when the time comes for you to lay your work aside the highest praise that can be given to you will be that you have worthily upheld the high traditions of your own school and the dignity of the most gentle profession.

